# A Study on the Negative Friction Mechanisms in Piles Within Recycled Dredged Waste Fills

**DOI:** 10.3390/ma18163904

**Published:** 2025-08-21

**Authors:** Xiangyang Hou, Wei Sun, Yongle Chen, Xiaoli Yi, Yaohui Liu, Lulu Liu

**Affiliations:** 1 China Railway First Group Co., Ltd., Xi’an 716006, China; 2 School of Mechanics and Civil Engineering, China University of Mining and Technology, Xuzhou 221000, China; believeliululu@163.com

**Keywords:** green low-carbon filling materials, dredged waste fills, pile foundation, negative friction, neutral point, centrifugal test

## Abstract

Green and low-carbon filling materials, primarily composed of dredged waste fills, are commonly used in the foundation of coastal highways. These materials possess high water content and under-consolidation characteristics, which can lead to differential settlement between piles and the surrounding environment. However, mechanical models of negative friction in piles within recycled dredged waste fills are insufficiently developed and presented. A mechanical model for the negative friction of a single pile in a composite foundation, consisting of dredged waste fills and other materials, is established based on the load transfer method. Through centrifugal model testing and numerical simulations, the development of negative friction and the migration pattern of the neutral point are analyzed and clarified. The results show that the theoretical model based on improved transfer function can effectively predict the neutral point position and negative friction value (average relative error < 6.5%). The theoretical analysis and experimental results indicate that the downward load due to negative friction increases nonlinearly. The loading strength exhibits a clear relationship with the consolidation process. Additionally, the dynamic evolution of the neutral point position is strongly correlated with consolidation of dredged fills. The size of pile foundation significantly influences the distribution of negative friction. Results show that the increment in negative friction for a pile with a 1.05 m diameter is 7.3% higher than that for a pile with a 1.5 m diameter. Smaller-diameter piles are more susceptible to negative friction due to the higher friction strength per unit area. The negative frictional resistance will enter a stable period after 50 months of settlement. The investigation can provide significant references for the design of pile foundations in areas with reclaimed materials, improving the stability and safety of pile foundations in practical engineering.

## 1. Introduction

In the context of the development of low-carbon transportation, recycled filling materials, primarily composed of hydraulic fill and dredged waste fills, are widely utilized in coastal highway subgrade widening. Coastal composite foundations, primarily composed of dredged waste fills and natural silt deposit, are prone to significant differential deformation. This susceptibility arises from the inherent properties of the fill, including high water content and highway loads [1,2,3]. The resulting pile–ground displacement difference induces negative friction. The negative friction substantially reduces pile foundation bearing capacity and contributes to structural issues. These issues represent typical performance risks in engineering applications utilizing such low-carbon, reclaimed materials [4,5]. Therefore, understanding the mechanism of pile–ground interaction within reclaimed material composite foundations is essential.

The engineering behavior of dredged waste fills, a key component in these composite foundations, is intrinsically linked to their fundamental material properties. Significant research efforts have been dedicated to characterizing the physical, chemical, and mechanical properties of dredged materials, including their geotechnical classification, compressibility, permeability, and consolidation characteristics [6,7]. For instance, Chen et al. [8] identified region-specific characteristics of Guangdong dredged fills, revealing their quartz mineralogy and agglomerated particle morphology. Studies have also investigated the influence of factors, such as mineralogy, organic content, salinity, and initial water content, on the long-term stability and deformation behavior of these fills [9,10]. For example, Lei et al. [11] investigated the effects of alkaline chemical conditions on the intergranular cementation and shear strength of dredged waste fills. Bao et al. [12] identified clay content as a critical factor governing consolidation properties under low-pressure conditions (<100 kPa), significantly influencing optimal stabilization timing for dredged fills. Understanding these material-specific attributes is crucial for predicting their performance as foundation materials.

Building upon the material properties of dredged fills, the long-term consolidation settlement inherent to reclaimed materials emerges as a critical engineering concern. This consolidation process directly impacts pile foundation stability through induced negative skin friction, as the consolidation-induced ground settlement creates differential displacement at the pile–foundation interface. Extensive research has investigated negative friction mechanisms in single-layer consolidating foundations, including theoretical frameworks for pile–foundation interaction [13,14,15], field observations of load-transfer evolution [16,17,18], and laboratory validations of neutral point dynamics during consolidation [19,20]. For instance, Chiou et al. [21] examined the influence of structural loading on the development of negative skin friction in both friction single piles and friction-end-bearing single piles in consolidating ground. Zhang et al. [22] investigated the negative friction of pile groups in backfill sand with varying groundwater levels. Zhao et al. [23] conducted a scale model test on the negative friction of piles, considering loess collapsibility, to study the change and distribution of negative friction in pile foundations within collapsible loess areas. Their results indicate that negative friction causes the pile to move downward, significantly increasing the pile’s axial force. The settlement of foundation is a very slow process. The continuous settlement of foundation for decades is a very common phenomenon which brings great challenges to model tests and field tests. As a key bridge between theoretical analysis and engineering practice, centrifugal model testing offers distinct advantages in replicating the stress field and time-dependent deformation [24,25,26,27,28,29]. There is limited research on the mechanism underlying the change in negative friction of pile foundations during foundation settlement in centrifugal tests. Furthermore, most previous studies have concentrated on homogeneous soils such as collapsible loess. In contrast, studies focusing on composite foundations, particularly those composed of degree fills, remain relatively scarce [30,31,32,33]. Consequently, there is an urgent need to investigate the development of negative friction in such composite reclaimed foundations using centrifugal model tests.

This study optimizes the mechanical model of pile foundations in composite fill–silt deposit systems, based on the load transfer method. Through a combination of centrifugal test validation and numerical simulation analysis, the study systematically reveals the development of negative friction and the impact of pile diameter during the consolidation process. These findings provide theoretical support for designing pile foundations in coastal reclamation areas.

## 2. Materials and Methods

### 2.1. Improved Transfer Function Model of Pile–Ground Interface

#### 2.1.1. Simplified Load Transfer Model of Pile–Ground Interface

**Figure 1:**
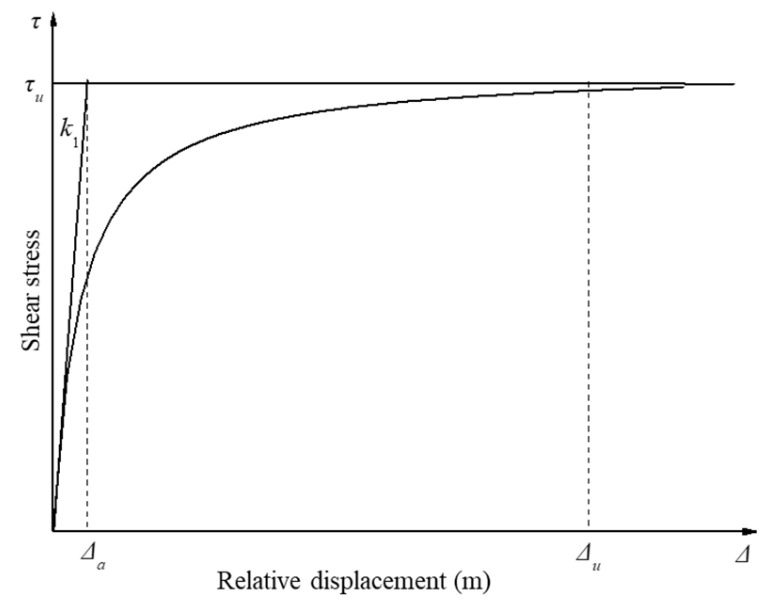
Relationship between shear stress and relative displacement of pile–ground interface.

In the pile–ground interface interaction, Wong et al. [34] assumed that the shear stress follows a hyperbolic relationship with the relative displacement, as illustrated in Figure 1. The x-coordinate is relative displacement (Δ), and the y-coordinate is shear stress (τ). The k1 is the change rate.

There are two methods to define the shear stiffness of pile–ground interface. Firstly, an empirical formula k_1_ = G/[r_0_ln(r_m_/r_0_)] is proposed by Randolph et al. [35]. G is the shear modulus of pile and ground, and r_0_ and r_m_ are the radius of pile and the influence radius of pile, respectively. Secondly, Alonso et al. [36] defined the ratio of ultimate shear stress and ultimate relative displacement. According to Terzaghi’s one-dimensional consolidation theory, the consolidation settlement at any time can be calculated as follows:

(1) $$w_{s}\left(z,t\right)=\frac{u_{0}L}{E_{s}}[1-\frac{z}{L}-\sum_{n=0}^{\infty }\frac{2}{M^{2}}e^{-M^{2}T_{v}}\cos(\frac{Mz}{L})]$$

where u_0_ is excess pore water pressure caused by additional load. T_v_ is dimensionless time factor. L is pile length, m; E_s_ is compressive modulus of ground; e is the void ratio; z is the depth, m; M = [(2n − 1)]π; and n = 1, 2, 3, …

**Figure 2:**
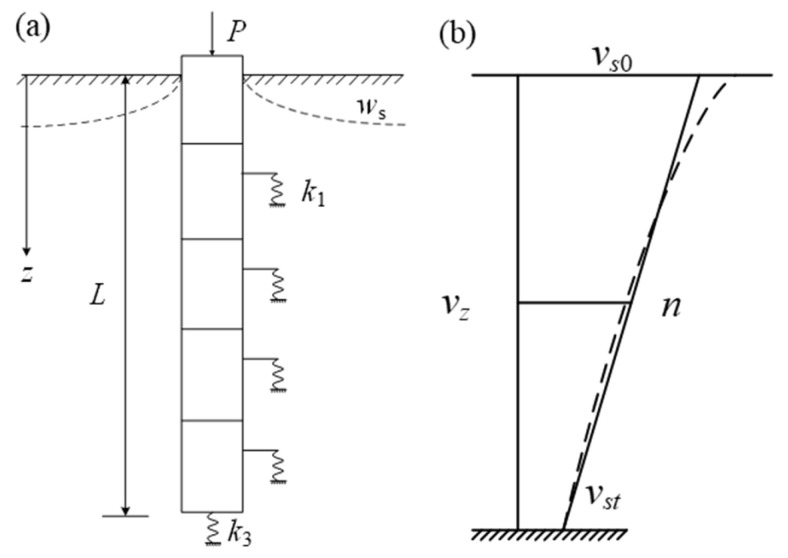
Calculation model of pile foundation by transfer function method. (**a**) Schematic diagram; (**b**) settlement of ground layer.

Zhou [37] proposed an analytical solution for the application of the negative friction of a single pile. The problem of negative friction caused by the loading of the filling materials is simplified. The pile–ground transfer model is shown in Figure 2. P is the external load, z is the depth, L is the length of the pile, ω_s_ is the consolidation settlement, k_1_ is the load transfer stiffness coefficient of the ground around the pile, and k_3_ is the supporting stiffness of the foundation at the end of the pile.

(1) The ground consolidation is supposed to develop linearly. As shown in Figure 2, v_s0_ is the final settlement of ground layer. v_st_ is the settlement of ground layer at any time. n is the settlement rate of ground layer.

(2) $$n=-\frac{dw}{dz}=\frac{v_{s\infty }-v_{st}}{L}$$

(2) It is assumed that the pile–ground transfer function is a linear elastic fully plastic relationship, as shown in Figure 1. k_1_ is the load transfer stiffness coefficient of the ground around the pile. As shown in Figure 1, Δa is the relative displacement corresponding to the k_1_. When the relative displacement of the pile and ground is greater than Δa, the friction resistance of the pile and ground is assumed to be a constant τ_u_.

(3) It is assumed that the pile tip is like a rigid block. Randolph et al. [35] used the Boussinesq formula (Equation (3)):

(3) $$w_{p}=\frac{P_{b}\left(1-\upsilon_{b}\right)}{4r_{0}G_{b}}$$

where w_p_ is pile tip displacement. P_b_ is pile bottom axial force. ν_b_ is Poisson’s ratio of ground under pile. r_0_ is pile radius. G_b_ is shear modulus of ground under pile.

The pile tip reaction is assumed to be linear elastic. k_3_ is the pile tip reaction coefficient, kN/mm.

(4) $$k_{3}=\frac{4r_{0}G_{b}}{1-\upsilon_{b}}$$

The bearing capacity of single pile is solved by the transfer function method. The pile is divided into some elastic elements, and the relationship between pile and ground is solved by using the above basic assumptions.

#### 2.1.2. Calculation Model of Negative Friction of Single Pile

Due to the low degree of consolidation of the degree fills, the relative displacement of the pile is large. Then the fills at the top of the pile enter the plastic stage quickly. Assuming that the depth of plastic deformation is h, Equation (5) can be obtained from the equilibrium conditions:

(5) $$\left\{\begin{array}{c} EA\frac{d^{2}w_{z1}}{dz^{2}}=k_{1}z_{u}\;\;0<z\leq h\\ EA\frac{d^{2}w_{z2}}{dz^{2}}=k_{1}w_{sz}\;\;h<z\leq L \end{array}\right.$$

where w_z_ is displacement of pile section, w_sz_ is pile–ground relative displacement, E is the Young’s modulus of the pile material, A is the cross-sectional area of the pile, z is the depth coordinate, L is the total length of the pile, and k_1_ is the shear stiffness coefficient of foundation.

When z = h, Equation (6) can be acquired:

(6) $$w_{z1}=w_{z2},EA\frac{dw_{z1}}{dz}=EA\frac{dw_{z2}}{dz}$$

The boundary conditions are shown in Equation (7). N_z_ is the axial internal force of the pile at depth z, P is the vertical load of the pile top, k_3_ is the end resistance stiffness coefficient of the foundation at the pile tip, and ω_sz_ is the vertical displacement at the pile tip.

(7) $$\left\{\begin{array}{c} Pile\;top\;boundary\;{\left.N_{z}\right|}_{z=0}=EA{\left.\frac{dw_{z1}}{dz}\right|}_{z=0}=P\\ Pile\;tip\;boundary\;{\left.N_{z}\right|}_{z=L}=EA{\left.\frac{dw_{z2}}{dz}\right|}_{z=L}={\left.-k_{3}w_{sz}\right|}_{z=L} \end{array}\right.$$

According to the relationship between w_z_, w_s_z, and v_z_, Equation (8) can be acquired:

(8) $$\frac{dw_{z}}{dz}=\frac{dw_{sz}}{dz}-n$$

Equations (5)–(8) can be simplified as Equations (9) and (10):

(9) $$\left\{\begin{array}{c} \frac{d^{2}w_{sz1}}{dz^{2}}=\frac{k_{1}\tau_{u}}{EA}\;\;0<z\leq h\\ \frac{d^{2}w_{sz2}}{dz^{2}}=\frac{k_{1}w_{sz2}}{EA}\;\;h<z\leq L\\ \frac{dw_{sz1}}{dz}-{\left.n\right|}_{z=0}=\frac{P}{EA}\\ \frac{dw_{sz2}}{dz}-{\left.n\right|}_{z=L}=-\frac{k_{3}}{EA}{\left.w_{sz2}\right|}_{z=L}\\ {\left.w_{sz1}\right|}_{z=h}={\left.w_{sz2}\right|}_{z=h},{\left.\frac{dw_{sz1}}{dz}\right|}_{z=h}={\left.\frac{dw_{sz2}}{dz}\right|}_{z=h} \end{array}\right.$$

(10) $$\left\{\begin{array}{cc} w_{sz1}\left(z\right)=\frac{1}{2}Gz^{2}+\alpha \zeta z+B & \left(0<z\leq h\right)\\ N_{1}\left(z\right)=EA\left(Gz+\alpha \zeta -n\right) & \left(0<z\leq h\right)\\ w_{sz2}\left(z\right)=C\sinh\alpha z+D\cosh\alpha z & \left(h<z\leq L\right)\\ N_{2}\left(z\right)=EA\left(C\alpha \cosh\alpha z+D\alpha \sinh\alpha z-n\right) & \left(h<z\leq L\right) \end{array}\right.$$

Definitions of A, B, and C are shown as follows:

$$\left\{\begin{array}{c} C=\frac{1}{\alpha }\cdot \frac{Gh\sinh\beta +\alpha \zeta \sinh\beta -n\sinh\alpha h+Gh\gamma \cosh\beta +\alpha \zeta \cosh\beta \gamma }{\sinh\beta \cosh\alpha h-\cosh\beta \sinh\alpha h-\gamma \sinh\beta \sinh\alpha h+\gamma \cosh\beta \cosh\alpha h}\\ D=-\frac{1}{\alpha }\cdot \frac{Gh\cosh\beta +a\zeta \cosh\beta +Gh\gamma \sinh\beta -n\cosh\alpha h+\alpha \zeta \gamma \sinh\beta }{\sinh\beta \cosh\alpha h-\cosh\beta \sinh\alpha h-\gamma \sinh\beta \sinh\alpha h+\gamma \cosh\beta \cosh\alpha h}\\ B=C\sinh\alpha h+D\cosh\alpha h-\frac{1}{2}Gh^{2}-\alpha \zeta h \end{array}\right.$$

where $\alpha =\sqrt{\frac{k_{1}}{EA}}$, $\beta =\alpha L=L\sqrt{\frac{k_{1}}{EA}}$, $\gamma =\frac{k_{3}}{EA\alpha }$, and $\zeta =\frac{n}{\alpha }+\frac{P}{EA\alpha }$.

When the relative displacement of pile is zero, it is the neutral point of pile foundation. Above the neutral point is negative friction and below is positive friction. When w_sz2_(z) = 0, the neutral point position can be obtained:

(11) $$z=\frac{1}{\alpha }\log\frac{\sqrt{\left|C^{2}-D^{2}\right|}}{C+D}$$

From Equation (11), it is evident that the position of the neutral point is influenced by several factors, including the characteristics of the pile foundation, the properties of the ground, the pile–ground interaction, and the load applied at the pile top. Additionally, it is affected by the distribution of the layers and the depth of the plastic zone.

The axial force distribution of the pile, as derived above, depends on factors such as ground settlement and pile–ground stiffness. The pile–ground stiffness can be determined through both field and laboratory tests. However, due to the time-consuming and costly nature of field measurements, the depth of plastic deformation in the ground can be measured by centrifugal tests.

### 2.2. Centrifugal Model Test

#### 2.2.1. Test Equipment and Scheme

A simulation test was conducted to investigate the negative friction of a single pile induced by the self-weight consolidation of dredger fill, using the geotechnical centrifuge platform at Tongji University. The test model box, measuring 600 mm (length) × 400 mm (width) × 500 mm (height), is a closed steel container. Its side walls are reinforced with a 50 mm thick steel plate to effectively constrain lateral fill deformation during the test.

**Figure 3:**
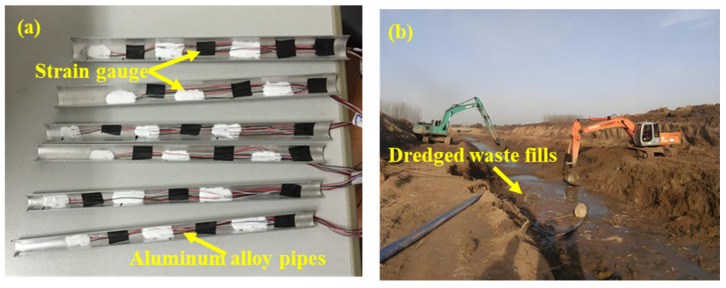
Pile foundation and dredger fills. (**a**) Physical model of pile foundation; (**b**) channel dredging.

The test model piles are made from aluminum alloy pipes with outer diameters of 20 mm, 16 mm, and 14 mm. Each pile has a standard length of 250 mm and a wall thickness of 1.03 mm (Figure 3). The pile cap is constructed from a 50 mm × 50 mm square aluminum alloy plate with a thickness of 5 mm, designed to simulate the load transfer interface at the top of the pile in practical engineering. Based on the actual formation distribution and the maximum load capacity of the centrifuge, the similarity ratio of the centrifugal test is 1:75.

The bearing layer at the pile end of the test model consists of a 70 mm thick saturated sand layer (with a particle size of approximately 2 mm), followed by a 50 mm silt layer and a 70 mm clay layer. This system is then covered by a 90 mm thick filling ground layer. The dredged fills used in the test were obtained from the river dredging site, as shown in Figure 3b. The remolded ground is prepared in a saturated state, and the model box is evenly filled with it using a layered weighing and compaction process. The centrifugal test, conducted under 75 g acceleration for 8 h, resulted in a consolidation degree of the foundation exceeding 90%. The physical and mechanical properties of each layer are provided in Table 1.

#### 2.2.2. Measurement System

The test monitoring system integrates multiple acquisition functions, including pile axial force, pile top settlement, foundation layer displacement, pore water pressure, and earth pressure. The axial strain of the pile is measured using seven sets of built-in half-bridge strain gauges (SGs), which are calibrated through a half-bridge temperature self-compensation circuit under 1 g conditions. Real-time displacement measurements of the pile top and ground layer settlement are recorded using a linear displacement sensor (LVDT). Earth pressure cells (PTs) and pore water pressure gauges (PKs) are installed at predetermined depths.

**Figure 4:**
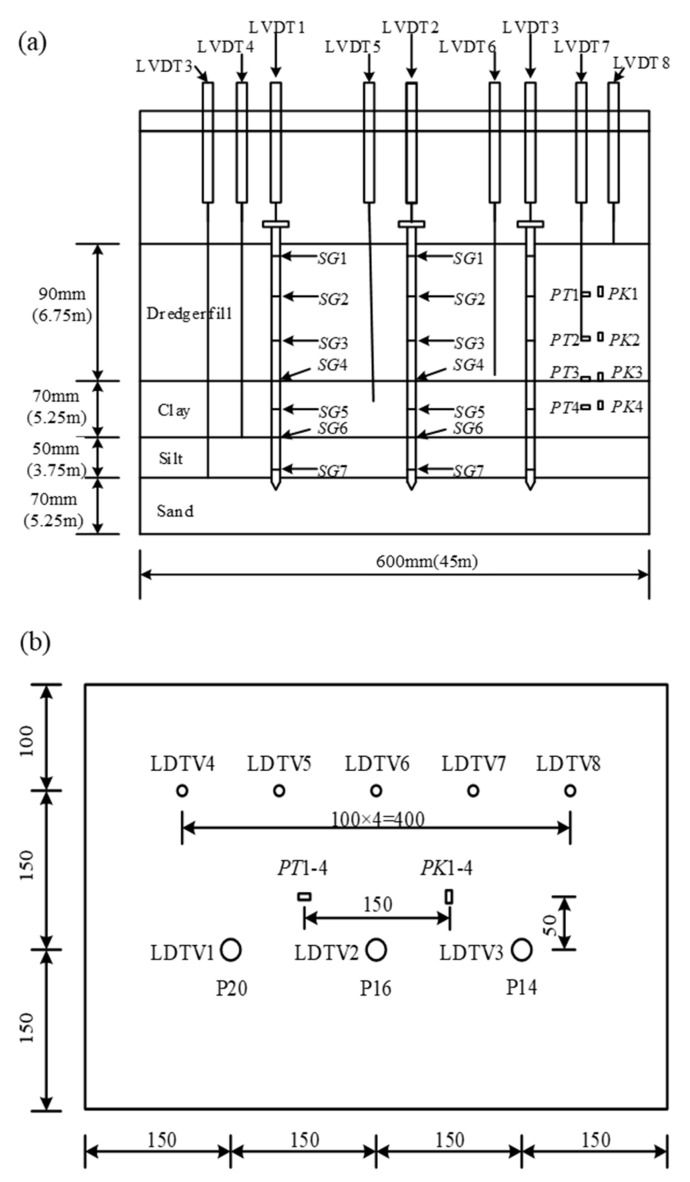
Device layout diagram for centrifugal test. (**a**) Sectional view; (**b**) top view (unit: mm).

For implementation, the aluminum alloy pipe pile is equipped with a strain gauge array, evenly distributed on the inner wall after longitudinal axial dissection. The gauges are encapsulated and protected with epoxy resin to form a complete pile body. The built-in wiring layout effectively minimizes ground disturbance around the pile. Additionally, the pile side surface is roughened with sandpaper to replicate the actual pile–ground interface friction characteristics. The ground layer settlement monitoring system consists of a φ40 mm aluminum alloy settlement plate and a φ6 mm rigid guide rod, arranged in a spatial array. The spatial positioning is shown in Figure 4. The units for distances in Figure 4 are in millimeters (mm). To help relate the foundation thickness in the model to the actual thickness in the project, the model’s soil thickness was labeled alongside its corresponding engineering thickness. The similarity ratio of the centrifugal test is 1:75. For instance, the dredger fill thickness in the model is 90 mm, which, according to the similarity theorem, corresponds to 6.75 m in practical engineering. This is indicated as 90 mm (6.75 m) in the figure. Additionally, the diagram includes the following instruments: LVDT (linear variable differential transformer) for displacement measurement, PT (pore water pressure transducer), PK (earth pressure transducer), and SG (strain gauge).

#### 2.2.3. Procedure of Centrifugal Test

**Figure 5:**
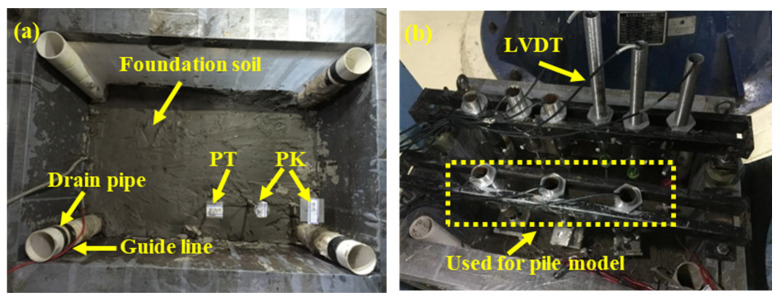
Pile–ground model: (**a**) foundation; (**b**) installation of device.

Firstly, the centrifuge was operated continuously for 8 h under a 75 g acceleration during which pore water pressure, earth pressure, and surface settlement evolution were monitored simultaneously. Loading was stopped once the consolidation degree of the clay layer reached 90%. Following this, under 1 g conditions, three model piles were installed. The diameters of model piles were 14 mm, 16 mm, and 20 mm, respectively. The pile end penetrated the sand layer to a depth of 1.5 times the pile diameter, and the pile foundation was driven into the specified position at a constant rate of 0.05 mm/s using a piling device (as shown in Figure 5). The stratified backfill ground was compacted to the design density, and the pile cap was subjected to the predetermined load for 30 min. The centrifuge was then restarted, maintaining the 75 g acceleration for another 8 h during which dynamic responses, including pile top displacement, pile body strain, ground stress, and layered settlement, were continuously monitored.

### 2.3. Numerical Simulation

#### 2.3.1. Parameter Settings for the Numerical Simulation

The FLAC analysis software (FLAC3D 5.0) is utilized in this study. The program is based on the Lagrangian algorithm and a hybrid discretization technique, which effectively simulates the three-dimensional elastoplastic response and large deformation characteristics of geotechnical materials.

The numerical layer consists of a dredged fill–clay–silt sequence. The 5.25 m thick silty sand layer (saturated, dense, and low compressibility) serves as the pile-end-bearing layer, while the 3.75 m thick sandy silty ground layer (saturated, medium density, and medium compressibility) and the 5.25 m thick clay layer form the primary compression layers. The model parameters include three pile diameter configurations: 1.05 m, 1.20 m, and 1.50 m. The load gradient applied to the top of the pile ranges from 630 kN to 1600 kN, increasing in intervals of 800 kN. The thickness of the filling materials is set in three series: 6.75 m, 9.75 m, and 12.75 m, while the geometric dimensions of the pile cap are fixed at 3 m × 3 m × 1 m. The physical and mechanical properties of each ground layer are determined from laboratory tests and field monitoring data, with specific values provided in Table 2.

#### 2.3.2. Pile–Ground Numerical Calculation Model

1. Basic assumptions

To simplify the calculation model, the following assumptions are made.

(1) The pile is considered an ideal isotropic elastic material, while the ground is modeled as an elastic-plastic material that satisfies the Mohr–Coulomb yield criterion.

(2) The particles are assumed to be homogeneous and fully saturated, with both the particles and water treated as incompressible.

(3) The deformation of each ground layer is assumed to be fully coordinated.

(4) The filling load is applied instantaneously as a single event.

2. Model description

**Figure 6:**
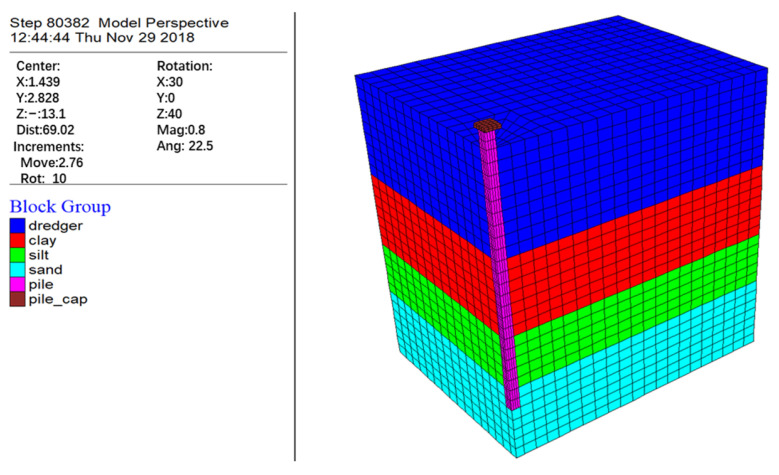
Grid division of geometric model.

In the numerical model, the pile–cap system is represented by an isotropic elastic constitutive model, while the ground medium follows the Mohr–Coulomb elastoplastic yield criterion. The Coulomb friction slip mechanism is introduced at the pile–ground contact interface. The self-weight consolidation process of the filling ground is modeled using fluid–solid coupling seepage mechanics. Based on the principle of symmetry, a quarter-scale reduced model is constructed as the calculation domain. The geometric model, meshing, and model dimensions are shown in Figure 6.

3. Boundary conditions

The boundary conditions of the model are defined as follows: the top boundary is a free-drainage boundary, the lateral boundaries impose a normal displacement constraint, and the bottom boundary applies a horizontal fixed constraint along with a vertical sliding constraint mechanism. The surface is subjected to a free-water-pressure boundary, while the global impermeable boundary condition is enforced. The bottom sliding bearing is modeled to exhibit mechanical response characteristics, allowing vertical displacement release and horizontal displacement locking through the contact element.

4. Loading sequence

First, the initial in situ stress field of the undisturbed foundation is established. Next, the pile foundation system is implanted, and the pile top load is applied. Finally, the graded loading process of the filling materials is implemented. The actual filling condition is simulated by applying an axisymmetric vertical load sequence to the ground. Through quasi-static equilibrium calculations of the pile–ground system, the entire evolution of the negative friction on the single pile is numerically reproduced, relying on parameterized control of the stacking boundary conditions.

## 3. Results and Discussion

### 3.1. Results and Analysis for the Centrifugal Model Test

According to the results of the centrifugal test and similarity ratio, the development of pore water pressure, ground layer settlement, pile top displacement, and pile axial force is calculated.

#### 3.1.1. Analysis of Settlement Development

**Figure 7:**
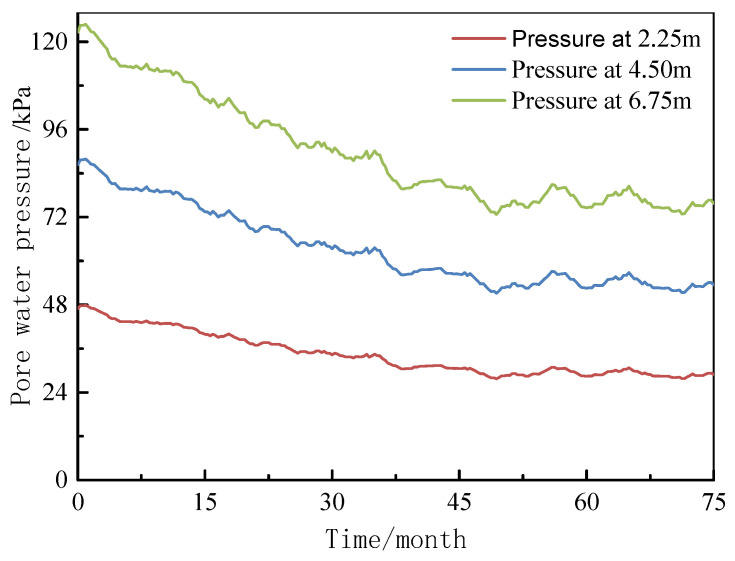
Variation curve of pore water pressure with time.

The pore water pressure measured at various depths is shown in Figure 7. As consolidation time increases, the pore water pressure decreases. A more significant reduction occurs within the first 30 months, after which the rate of decrease slows, stabilizing around 45 months. Ultimately, the pore water pressure reduces to approximately 88.84%, primarily due to the dissipation of excess pore water pressure in the fill material.

**Figure 8:**
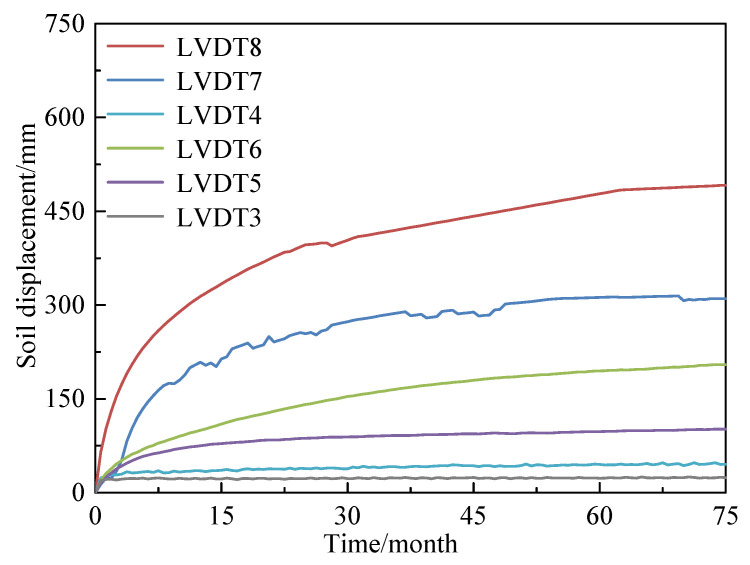
Layered settlement of ground changes with time.

The ground settlement development is shown in Figure 8. The settlement of each ground layer generally reached a stable state within 75 months. The total settlement accumulated to 491.1 mm, with the sand layer, characterized by high permeability, undergoing rapid settlement during the initial stage of heaped filling (approximately 84.56% of the total settlement in the sand layer). Afterward, the settlement rate remained relatively constant. The silt layer completed most of its settlement within 5 months of construction, while the clay and filling layers experienced a faster settlement rate during the first 15 months. This stage accounts for about two-thirds of the total settlement. Notably, after 45 months, the entire ground system entered a slow consolidation phase, with the settlement rate decreasing to 15–20% of the initial rate. Analysis of the ground characteristics reveals that the sand layer has good permeability, allowing for the rapid dissipation of excess pore water pressure after loading. This is a key factor in the early completion of consolidation in the sand layer. In contrast, the settlement processes of the clay and dredged fill layers exhibit clear time-varying characteristics due to the slower rate of drainage consolidation.

**Figure 9:**
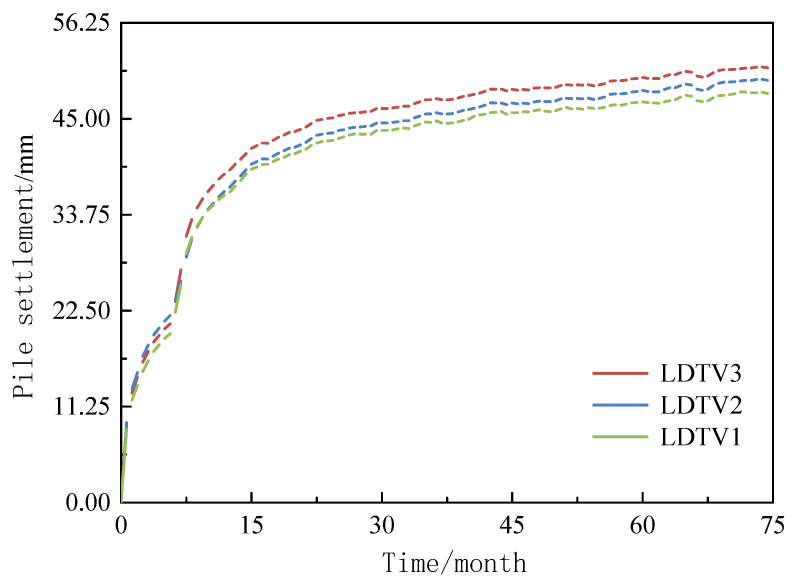
Relationship between pile displacement and time.

When the pile diameters are 1.05 m, 1.2 m, and 1.5 m, the settlement responses of a single pile are generated under the combined effects of ground self-weight consolidation and pile top loads of 1103.16 kN, 1260.77 kN, and 1575.96 kN, respectively. The settlement behavior follows a pattern similar to that of the ground, exhibiting typical two-stage characteristics: rapid development in the early stage and gradual stabilization in the later stage (see Figure 9). Notably, the settlement corresponding to the larger pile diameters shows a decreasing trend. The final settlements for pile diameters of 1.05 m, 1.2 m, and 1.5 m are 51.05 mm, 49.61 mm, and 48.08 mm, respectively. It is important to note that despite the increase in load values with larger pile diameters (1.05 m pile diameter corresponds to 1103.16 kN, while the 1.5 m pile diameter reaches 1575.96 kN), the actual settlement decreases by approximately 5.8%. This is attributed to the stress diffusion effect resulting from the larger pile bearing area, suggesting that a reasonable increase in pile diameter can effectively control foundation settlement.

#### 3.1.2. Axial Force of Pile Changing with Depth

**Figure 10:**
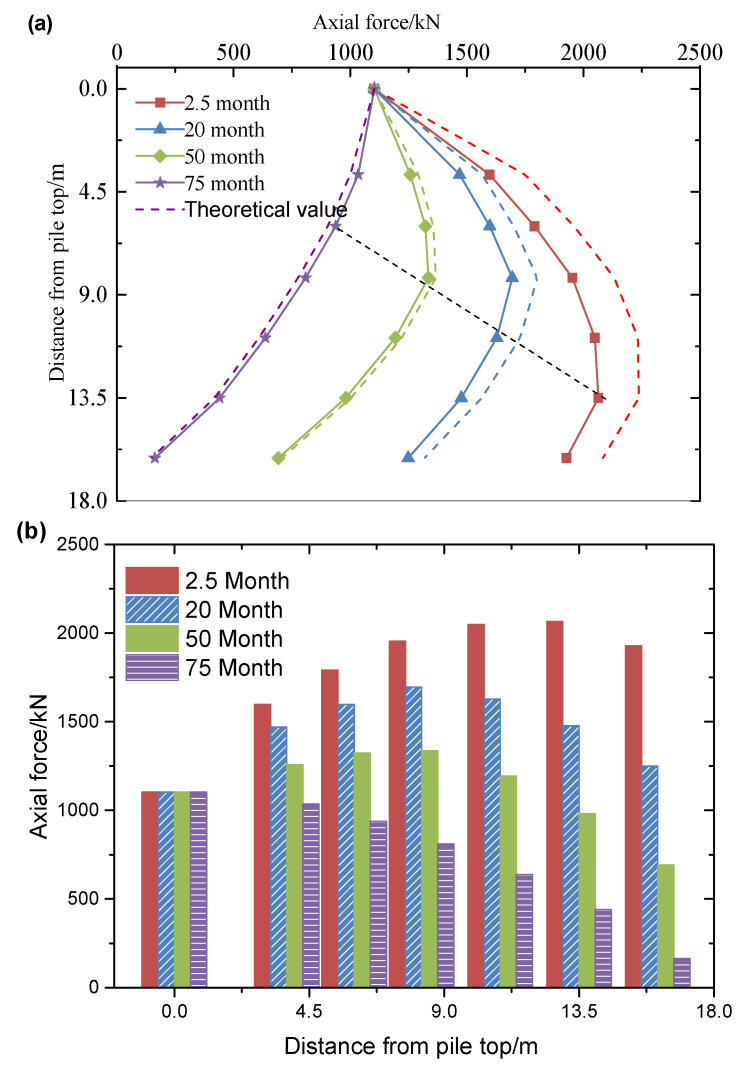
The development of axial force at different positions of pile with 1.05 m diameter: (**a**) line chart; (**b**) bar chart.

**Figure 11:**
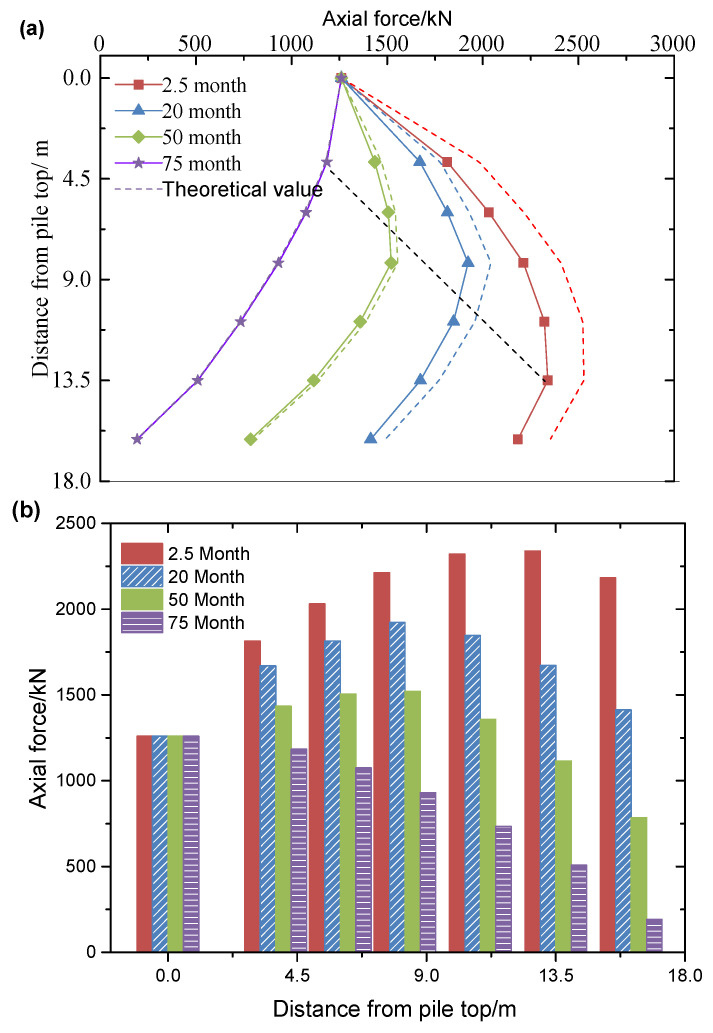
The development of axial force at different positions of pile with 1.2 m diameter: (**a**) line chart; (**b**) bar chart.

**Figure 12:**
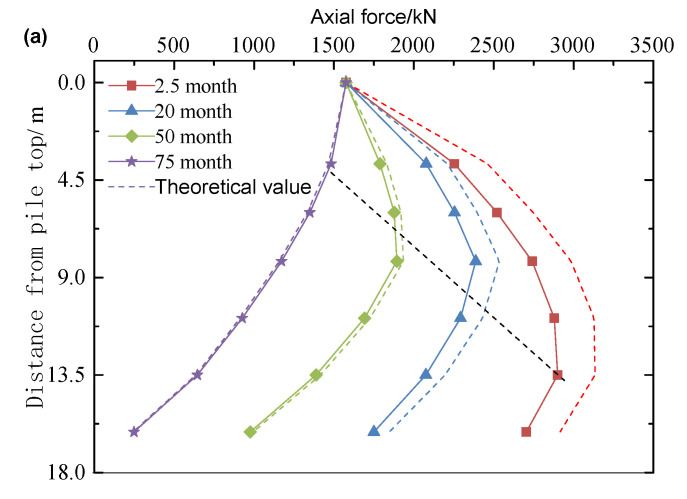

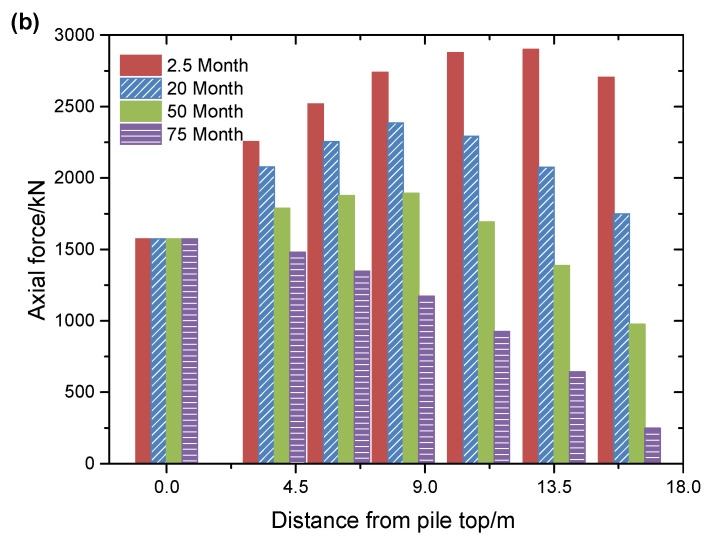
The development of axial force at different positions of pile with 1.5 m diameter: (**a**) line chart; (**b**) bar chart.

The ground layers are sequentially distributed from top to bottom, consisting of dredged fills, clay, silt, and medium-fine sand. Prototype data were obtained through centrifugal model testing. Figure 10, Figure 11 and Figure 12 compare the measured values (solid line) with the theoretical calculation values (dotted line) for the axial force in the pile. The test results indicate that the axial force of the pile increases nonlinearly with consolidation time, with a significantly higher rate of development in the early stages compared to the later stages. Additionally, the neutral point (the peak position of the axial force) continues to shift upward during consolidation, suggesting that the pile–ground interaction exhibits significant aging characteristics. The theoretical calculations align well with the measured results, with the specific error showing a regular trend over consolidation time: the error is 8.71% at 2.5 months, decreases to 6.56% at 20 months, further reduces to 2.38% at 50 months, and shows a negative deviation of −4.79% at 75 months. In the early stages of consolidation, the theoretical value is higher than the measured value (with a maximum deviation of 8.71%), while in the later stages, it is lower (with a maximum deviation of −4.79%). Since negative friction predominantly affects the early stage of consolidation, the theoretical calculations tend to be more conservative than the actual results. These conservative calculation characteristics provide a reliable theoretical basis for the design of engineering pile foundations.

When pile foundations with diameters of 1.05 m, 1.2 m, and 1.5 m are subjected to initial loads of 1103.16 kN, 1260.77 kN, and 1575.96 kN, respectively (Table 3), the development of negative friction exhibits a significant size effect. Specifically, under identical consolidation conditions, smaller-diameter pile foundations demonstrate a stronger negative friction response. For instance, after 2.5 months of consolidation, the measured negative friction of the 1.05 m pile reaches 291.40 kN, approximately 3.6% higher than the 281.20 kN measured for the 1.5 m pile. This phenomenon arises from the fact that the negative friction strength at unit area increases as the pile diameter decreases. This leads to a greater pull-down load on smaller piles during the self-weight consolidation of the fill material. Although the initial load on the larger diameter pile foundation is greater (the load on the 1.5 m pile is 42.8% higher than that on the 1.05 m pile), the increase in negative friction is smaller. This supports the engineering principle that a reasonable increase in pile diameter can effectively reduce the influence of negative friction.

#### 3.1.3. Variation Law of Neutral Point with Consolidation Time

As shown in Figure 10, Figure 11 and Figure 12, the position of the neutral point exhibits dynamic evolution throughout the consolidation process. The measured data indicate that the neutral point lies between 0.51 L and 0.84 L (where L is the pile length), while the theoretical prediction ranges from 0.54 L to 0.87 L. The spatial distribution of both sets of data is highly consistent, with an average relative deviation of only 5.88%. As consolidation time progresses, the neutral point consistently shifts upward. This spatial–temporal evolution is clearly reflected in both the measured and theoretical curves. The prediction accuracy of the theoretical model in the later stages of consolidation (beyond 0.8 L) is approximately 12% higher than in the early stages, thereby confirming the engineering applicability of the neutral point calculation method that accounts for time dependence.

### 3.2. Results of Numerical Simulation

**Figure 13:**
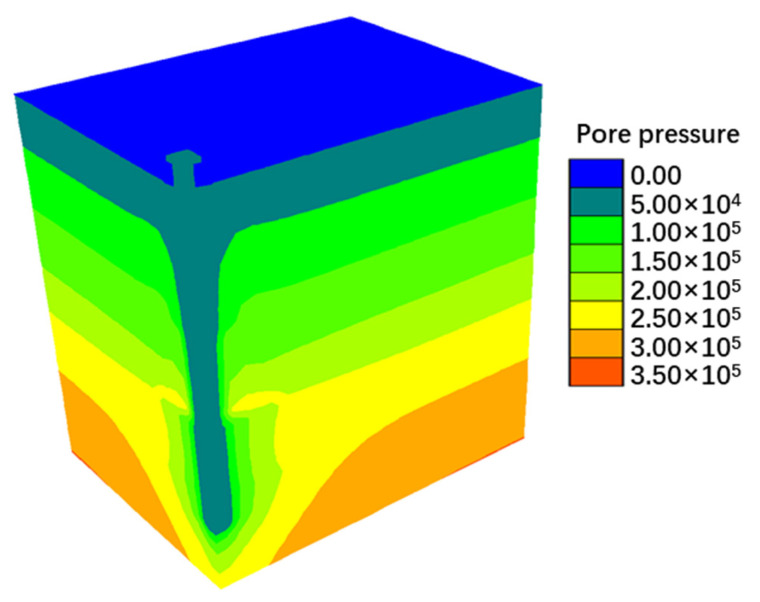
Pore water pressure nephogram.

**Figure 14:**
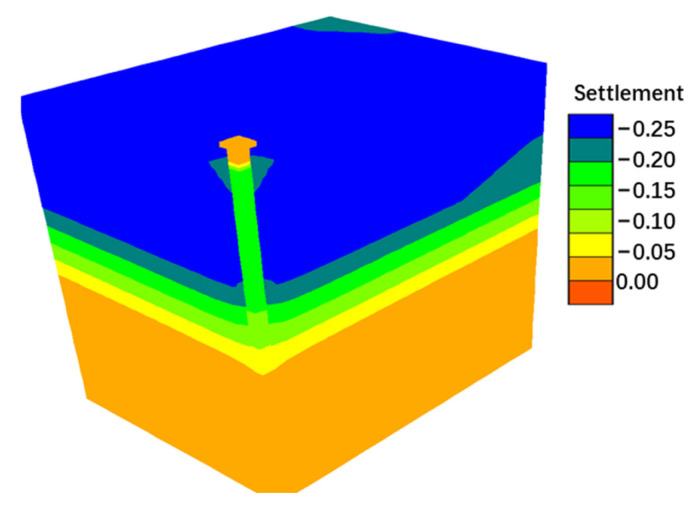
Settlement cloud chart.

**Figure 15:**
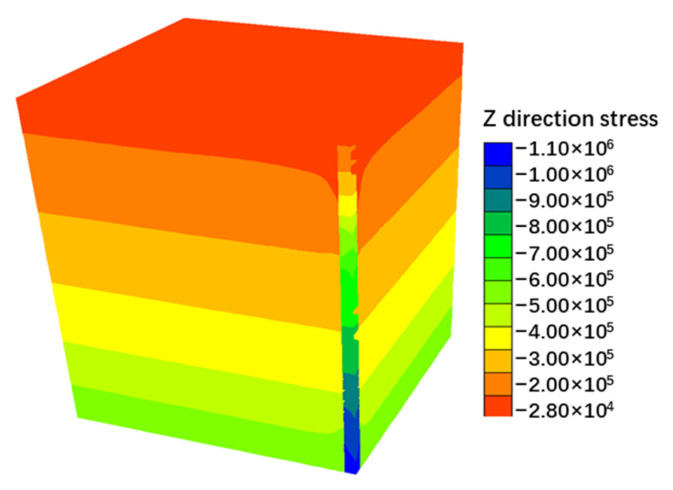
z direction stress cloud chart.

The numerical simulation results successfully replicate the spatial distribution characteristics of the pore water pressure field, settlement field, and z-direction stress field (as shown in Figure 13, Figure 14 and Figure 15). The evolution data for earth pressure, pore water pressure, and settlement at monitoring points (3.75 m, 6.75 m, and 9.75 m) over consolidation periods of 2.5, 5, 10, 20, 30, 40, 50, 60, 70, and 75 months are systematically recorded. Additionally, the stress response time history curves for pile monitoring sections (0 m, 3.75 m, 6.0 m, 8.25 m, 10.875 m, 13.5 m, and 16.125 m) are obtained concurrently.

**Figure 16:**
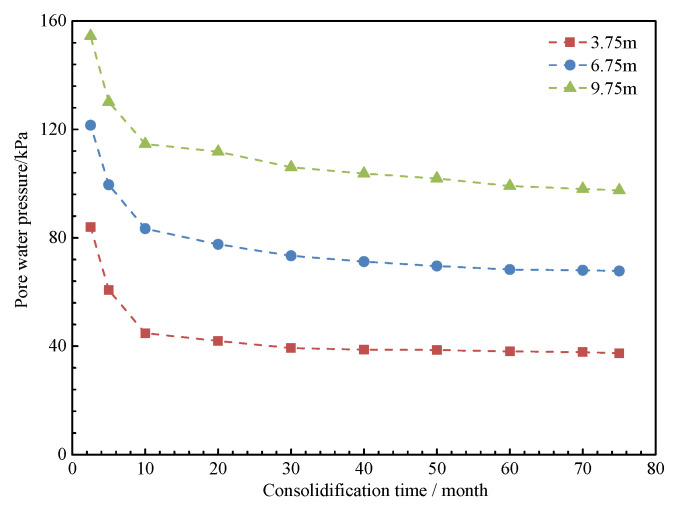
Pore water pressure at different consolidation times.

**Figure 17:**
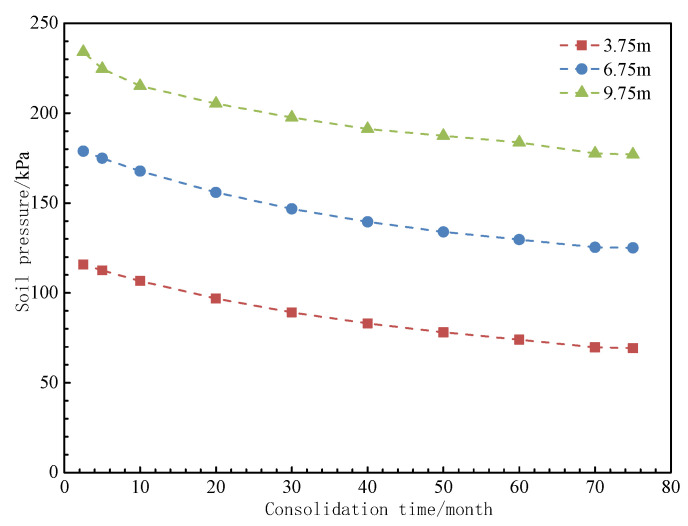
Earth pressure at different consolidation times.

**Figure 18:**
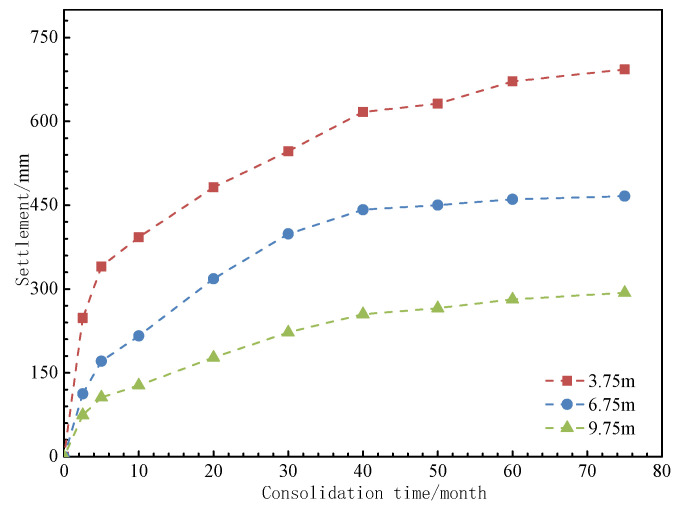
Settlement at different consolidation times.

**Figure 19:**
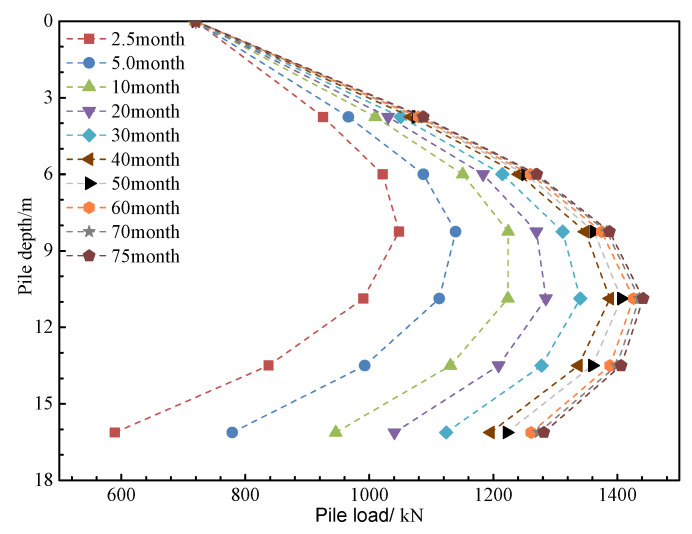
Pile load at different consolidation times.

The numerical simulation results provide insights into the evolution of pore water pressure, earth pressure distribution, and settlement characteristics during the consolidation stages at 2.5, 5, 10, 20, 30, 40, 50, 60, 70, and 75 months. The stress response values for monitoring sections at depths of 0 m, 3.75 m, 6.0 m, 8.25 m, 10.875 m, 13.5 m, and 16.125 m are recorded. The evolution patterns of these parameters are presented in Figure 16, Figure 17, Figure 18 and Figure 19.

As shown in Figure 16 and Figure 17, the initial excess pore water pressure of 42.5 kPa in the filling ground layer exhibits a phased dissipation pattern during self-weight consolidation drainage. The dissipation rate is 0.32 kPa/day during the first 10 months, with a total dissipation of 78.4%. The dissipation rate then decreases to 0.05 kPa/day over the next 65 months, eventually leading to complete dissipation by 75 months. As pore water is discharged, the earth pressure gradually decreases from an initial value of 152.6 kPa to a stable value of 128.4 kPa, representing a 15.9% reduction.

As shown in Figure 18, during the discharge of excess pore water, the self-weight settlement of the fill material follows a time-varying pattern similar to the dissipation of pore water. The settlement rate is initially faster in the first 10 months and then gradually slows down, eventually stabilizing. The total settlement of the 3.75 m thick filling ground layer at the top reaches 226.57 mm, while the total settlement of the 3.0 m thick ground layer at the bottom is 172.94 mm.

As shown in Figure 19, the negative friction effect on the pile, caused by the self-weight consolidation settlement of the fill material, results in the total load on the pile significantly exceeding the initial load at the pile top. The development pattern indicates a much higher growth rate during the first 10 months, followed by a slower increase, with the load reaching a stable state after approximately 60 months. The simulation results show that the maximum load on the pile is 1441.58 kN (721.58 kN greater than the initial pile top load, representing a 100.21% increase). The peak load is distributed between depths of 8.25 m and 10.875 m, corresponding to a range of 1.5 m to 4.125 m below the top surface of the original foundation, with a relative buried depth ratio ranging from 0.167 to 0.458.

Table 4 summarizes the key characteristics of several studies. Overall, existing research predominantly focuses on analyzing the negative friction of pile foundations in single soils (typically natural undisturbed soils) using a single method such as scale model tests or numerical simulations. In contrast, studies investigating the formation of new foundations using green, low-carbon materials remain limited. This study employs a combination of centrifugal model tests, theoretical calculations, and numerical simulations to explore the mechanisms underlying the negative friction of pile foundations in composite soils. Notably, foundation settlement is often a slow process, taking decades to occur, which presents significant challenges for both model testing and field monitoring. In this context, the centrifugal model test offers a solution by overcoming the limitations of long-term settlement simulation and enhancing the accuracy of prototype simulations through its time-compression advantage.

## 4. Conclusions

This study develops a calculation model for pile negative friction in coastal composite foundations. These foundations utilize solid waste-based green low-carbon filling material combined with silt deposit. Through collaborative verification with centrifugal model tests and numerical simulations, the spatial and temporal evolution of negative friction in composite foundations is revealed. The main conclusions are as follows:

(1) The calculation results of the theoretical model show good agreement with the centrifugal test and numerical simulation data, with an average relative error of less than 6.5%. This confirms the engineering reliability of the improved calculation method. The comparison between experimental and theoretical results for the axial force of the pile reveals that the maximum deviation occurs in the initial consolidation stage (approximately 8.7% at 2.5 months), and the deviation can decrease to 2.4% as consolidation progresses to 50 months.
(2) The geometric parameters of the pile significantly influence the distribution of negative friction. Under identical consolidation conditions, the increment in negative friction for a pile with a 1.05 m diameter is 7.3% higher than that for a pile with a 1.5 m diameter. This is primarily because the frictional resistance strength per unit area increases as the pile diameter decreases, making smaller-diameter pile foundations more susceptible to the adverse effects of pull-down load. In composite foundations composed primarily of dredged fills, the use of larger-diameter pile foundations is beneficial for reducing negative friction.
(3) The development of negative friction exhibits three distinct aging stages: a rapid rise period (0–10 months, with a growth rate of 0.85 kN/day), a slow growth period (10–50 months, with a growth rate of 0.12 kN/day), and a stable dissipation period (after 50 months). During the consolidation process, the position of the neutral point moves downward from 0.84 L (at 2.5 months) to 0.51 L (at 50 months). The rate of neutral point migration is negatively correlated with the degree of ground consolidation. In practical engineering, the settlement of dredged fills can be accelerated through consolidation techniques, such as preloading, to facilitate the rapid transition of the negative friction resistance of pile foundations into a stable dissipation phase.

The mechanical model based on the load transfer method effectively evaluates the negative friction in composite foundations, providing a valuable reference for the design of pile foundations in green and low-carbon filling materials composite subgrades.

## Figures and Tables

**Table 1:** Physical index parameters of foundation in centrifugal test.

Foundation	Dredger Fill	Clay	Silt	Sand
Thickness of the layer/m	5~14	5.25	3.75	5.25
Water content/%	32.2	43.3	30.4	26.6
Weight/(kN/m^3^)	18.5	17.4	18.6	19
Specific gravity	2.71	2.75	2.70	2.69
Void ratio		1.22	0.86	0.76
Liquid limit		46.4		
Plastic limit		25.0		
Plasticity index		21.3		
Liquidity index		0.84		
Cohesion/kPa	11	14	7	4
Internal friction angle/°	29	13	33	35
Compressibility coefficient	0.24	0.80	0.14	0.09
Compression modulus/MPa	8.58	2.94	14.51	21.59

**Table 2:** Physical and mechanical parameters in FLAC3D.

Parameter	Dredger Fill	Clay	Silt	Sand	Concrete
Dry density/(kN/m^3^)	13.99	11.98		15.01	24
Saturation density/(kN/m^3^)	18.5	17.4	18.6	19.0	/
Compression modulus/(MPa)	8.58	2.94	14.51	21.59	/
Poisson ratio	0.35	0.45	0.33	0.35	0.2
Cohesion/(kPa)	11	14	7	4	/
Internal friction angle/(°)	29	13	33	35	/
Shear modulus/(MPa)	4.77	2.58	11.91	14.94	1.92 × 10^4^
Bulk modulus/(MPa)	2.86	0.27	4.57	4.98	1.44 × 10^4^
Permeability coefficient/(m/s)	2.31 × 10^−11^	2.05 × 10^−12^	3.72 × 10^−10^	5.45 × 10^−9^	/
Porosity	0.68	0.55	0.46	0.43	
Biot’s coefficient	1	1	1	1	/
Biot modulus/(Pa)	2.94 × 10^9^	3.64 × 10^9^	4.35 × 10^9^	4.65 × 10^9^	/
Tangential stiffness of contact surface/(MPa/m)	17.17	5.88	36.01	41.36	/
Normal stiffness of contact surface/(MPa/m)	12.02	4.12	25.21	28.95	/
Modulus of elasticity (GPa)	/	/	/	/	30

**Table 3:** The development of the maximum negative friction and the neutral point position.

Consolidation Time/Month	Pile Diameter/m	Test Value of Negative Friction/kN	Theoretical Value of Negative Friction/kN	Measured Neutral Point/m	Theoretical Neutral Point/m
2.5	1.05	960.75	1134.08	13.5	13.98
	1.2	1078.58	1267.22	13.5	13.84
	1.5	1324.44	1558.30	13.5	13.65
20	1.05	591.72	699.55	8.25	9.56
	1.2	662.73	739.49	8.25	9.31
	1.5	811.83	959.80	8.25	9.08
50	1.05	232.43	264.48	8.25	8.68
	1.2	260.33	304.53	8.25	8.53
	1.5	318.89	358.62	8.25	8.45
75	1.05	−11.77	−28.6	/	/
	1.2	−20.59	−33.59	/	/
	1.5	−26.39	−40.04	/	/

**Table 4:** Summary of research methods for negative friction of pile foundation.

Number	Method	Foundation	Foundation Type	Author
1	Centrifugal test/theoretical Calculation/numerical test	Dredged fills and natural silts	Composite soil	This study
2	Scale model test	Collapsible loess	Homogeneous soil	Zhao [23]
3	Numerical test	Soft clay	Homogeneous soil	Chiou [21]
4	Model test	Sand	Homogeneous soil	Zhang [22]
5	Model test	Collapsible loess	Homogeneous soil	Chai [30]
6	Theoretical calculation	Soft clay	Homogeneous soil	Wu [32]

## Data Availability

The original contributions presented in this study are included in the article. Further inquiries can be directed to the corresponding author.
